# Alterations in peripheral blood immune cell profiles of neuromyelitis optica spectrum disorder across different phases and after B cell depletion therapy

**DOI:** 10.3389/fimmu.2025.1556259

**Published:** 2025-06-11

**Authors:** Xuying Wang, Ruoyi Guo, Zhichao Yao, Lu Liu, Zhen Jia, Xiujuan Song, Bin Li

**Affiliations:** ^1^ Department of Neurology, The Second Hospital of Hebei Medical University, Shijiazhuang, China; ^2^ Key Laboratory of Neurology (Hebei Medical University), Ministry of Education, Shijiazhuang, China; ^3^ Key Laboratory of Neurology of Hebei Province, Shijiazhuang, China; ^4^ Department of Neurology, Baoding No.1 Central Hospital, Baoding, China

**Keywords:** neuromyelitis optica spectrum disorder, b cell depletion therapy, peripheral blood immune cells, multiple sclerosis, myelin oligodendrocyte glycoprotein antibody disease

## Abstract

**Introduction:**

Peripheral blood immune cell profiles of neuromyelitis optica spectrum disorder (NMOSD) are still unclear under different disease states and after B cell depletion therapy. Moreover, NMOSD is often confused with multiple sclerosis (MS) and myelin oligodendrocyte glycoprotein antibody disease (MOGAD). The study aims to illustrate peripheral blood immune cell profiles of NMOSD under different disease states, after B cell depletion therapy, and compared with MS and MOGAD.

**Methods:**

This study included 76 NMOSD patients, 20 MS patients, and 12 MOGAD patients in the acute phase, along with 37 controls whose sex and age were matched with NMOSD patients. Forty-two of 76 patients with acute NMOSD were followed in remission, of whom 13, 15, and 11 patients received rituximab treatment, inebilizumab treatment, or conventional immunosuppressive therapies alone, respectively. The levels of diverse peripheral blood immune cells were measured by blood routine examination and flow cytometry. Distinctions among groups were analyzed using statistical methods.

**Results:**

Compared with controls and NMOSD patients in remission, there was an elevation in the levels of neutrophils, platelets, platelet/lymphocyte ratio, neutrophil/lymphocyte ratio, and systemic inflammation index in acute NMOSD patients, while a decline was observed in the levels of lymphocytes, eosinophils, basophils, CD3+ T cells, CD3+CD4+ T cells, and CD4/CD8 ratio. NMOSD had increased levels of platelets and platelet/lymphocyte ratio, and decreased levels of eosinophils, basophils, CD4/CD8 ratio, and CD3+CD4+ T cells compared with MS. NMOSD had decreased levels of eosinophils, basophils, and CD19+ B cells, along with elevated platelet/lymphocyte ratio compared with MOGAD. After rituximab treatment, not only did CD19+ B cell level decrease, but eosinophil counts also increased. After inebilizumab treatment, not only did CD19+ B cell level decrease, but also the ratios of CD3+ T cells and CD3+CD8+ T cells increased. The quantity and ratios of eosinophils in rituximab group surpassed those in inebilizumab group.

**Discussion:**

Peripheral blood immune cell profiles of acute NMOSD patients showed widespread distinctions compared to those of remission NMOSD patients, acute MS patients, acute MOGAD patients, and controls, as well as after differential therapies. Our findings provide clues to comprehensively understand the abnormality of the dynamic and integrated immune network in NMOSD, distinguish NMOSD from MS and MOGAD, and search for more effective and safe therapeutic targets.

## 1 Introduction

Neuromyelitis optica spectrum disorder (NMOSD), characterized by aquaporin-4 immunoglobulin G (AQP4-IgG), is a rare autoimmune disorder with an incidence rate of 0.039–0.73 per 100,000 person-years (1) and a striking female predominance (9:1 ratio). The median age at which individuals typically experience the onset of NMOSD is 40 years (2, 3). NMOSD is a relapsing and severe disorder leading to significant neurological disabilities, such as blindness and paralysis, or even death. Therefore, discovering the pathophysiology of NMOSD, with the aim of identifying more efficacious treatment modalities and enhancing patient prognosis, holds significant value.

AQP4-IgG is generated in B cells of peripheral blood, penetrates the central nervous system (CNS) via the damaged blood-brain barrier, and bonds to astrocyte endfeet, resulting in astrocyte destruction that ultimately leads to oligodendrocyte loss and demyelination (4). Overall, since AQP4-IgG originates peripherally, NMOSD is regarded as an autoimmune disease peripherally antibody-mediated and manifested in the CNS. A diversity of peripheral blood immune cells are essential for immune homeostasis. Nevertheless, abnormalities in peripheral blood immune cells can have catastrophic effects, disrupting immune homeostasis and potentially contributing to the onset of autoimmune diseases. Since the immune system is a dynamic and integrated immune network, a comprehensive analysis of diverse immune cells, rather than an isolated one, is more conducive to reflecting unique immunopathological processes. Previous studies have focused on a certain B cell subset or T cell subset of NMOSD patients (5–9). However, to our knowledge, few studies have comprehensively analyzed peripheral blood immune cell profiles of NMOSD patients, let alone comparing those between the acute phase and remission in the same patients.

For many years, conventional immunosuppressive therapies (CIT), including oral immunosuppressants and corticosteroids, have been employed to prevent relapse of NMOSD. However, 25-60% of patients continued to experience recurrent attacks (10). The rapidly emerging B cell depletion therapy (BCDT) has been a promising treatment for relapse prevention by depleting B cell populations (11, 12). But rarely, have the effects of BCDT and distinct effects between different B cell depleting drugs on other peripheral blood immune cells been explored.

NMOSD, multiple sclerosis (MS), and myelin oligodendrocyte glycoprotein antibody disease (MOGAD) are CNS demyelinating diseases. Despite divergent etiologies, pathophysiologies, therapeutic strategies, and prognoses, their clinical manifestations can sometimes be confounded, posing diagnostic challenges. Frequently, discrimination can be made between NMOSD and the other two conditions, MS and MOGAD, by the serologic test of AQP4-IgG with cell-based assay. Due to potential unavailability and ambiguity of the assay (13), there is a pressing need for indicators that could differentiate patients with NMOSD from MS and MOGAD patients. Comparative studies of NMOSD, MS, and MOGAD involving clinical characteristics (14), low abundance nervous system proteins (15) and complement 3 and complement 4 (16) in blood, cerebrospinal fluid biomarkers (17, 18), magnetic resonance imaging features (17, 19, 20), and optical coherence tomography markers (19) have been carried out. Yet, the contribution of peripheral blood immune cell profiles to the differential diagnosis is poorly understood.

In this research, we analyzed peripheral blood immune cell profiles of patients with NMOSD across disease states. Moreover, we elucidated the effects of BCDT on peripheral blood immune cell profiles. Additionally, we characterized distinctive peripheral blood immune cell profiles between MS, MOGAD, and NMOSD.

## 2 Materials and methods

### 2.1 Participants and groups

The study included 76 NMOSD patients, 20 MS patients, and 12 MOGAD patients hospitalized from September 1, 2022, to October 15, 2024, in the Neurology Department of the Second Hospital of Hebei Medical University and was approved by the Second Hospital of Hebei Medical University Ethics Committee(Approval No. 2021-R513).

NMOSD patients, MS patients, and MOGAD patients met the 2015 NMOSD diagnostic criteria (21), 2017 McDonald criteria (22), and 2023 MOGAD diagnostic criteria (23), respectively. All research subjects were Han Chinese from northern China and in the acute phase, which referred to a new, recurring, or worsening neurological deficit persisting for no less than 1 day and occurring within less than 30 days (24). According to whether they had received disease-modifying therapy (DMT) before onset, patients with acute NMOSD (n = 76) were classified as DMT-NMOSD (n = 18) and non-DMT-NMOSD (n = 58). In the non-DMT-NMOSD group, 22 patients were experiencing their first onset of the disease and had not yet initiated DMT. The remaining 36 patients in the non-DMT-NMOSD group did not receive DMT due to various reasons, including personal choices. Specifically, some patients opted not to initiate DMT due to concerns about potential side effects or a preference for alternative treatment approaches. Others were referred late to our specialized center, resulting in a delay in the initiation of DMT. Notably, none of the patients with acute MS (n = 20) or acute MOGAD (n = 12) in the study had received DMT before onset. The study excluded patients who had the following characteristics: (1) individuals younger than 14 years of age, (2) significant acute infection, (3) autoimmune thyroiditis, sjögren syndrome, systemic lupus erythematosus, and other autoimmune conditions, (4) concomitant malignancies, (5) serious hematologic disorders, (6) individuals had received intravenous methylprednisolone, plasma exchange, or other acute phase therapies before inclusion. Of 76 patients with acute NMOSD, 42 patients were followed no less than 1 month subsequent to the attack, forming the attack-remission paired cohort, which included remission NMOSD group (n = 42) and paired acute NMOSD group (n = 42). Of 42 patients in remission, a total of 15, 13, 2, 1, and 11 patients received inebilizumab, rituximab, tocilizumab, ofatumumab, or CIT alone, respectively. Patients treated with tocilizumab and ofatumumab were not analyzed due to the small sample size, while others were categorized into three groups, namely inebilizumab group (n = 15), rituximab group (n = 13), and CIT group (n = 11). Since both rituximab and inebilizumab belong to the category of BCDT, we combined the rituximab group and the inebilizumab group into a new group, which is also designated as the BCDT group (n = 58). Additionally, 37 controls (control group, Control), matched for sex and age with NMOSD patients, were recruited. These individuals were free from significant acute infections, autoimmune diseases, malignancies, and severe hematological diseases. Besides, all controls had not received any medications that could affect peripheral blood immune cells within the preceding three months. An informed consent form was obtained from every subject prior to participating.

### 2.2 Clinical data

Clinical data of participants, including sex, age, lesion location, the number of onsets, the time from last symptom to clinic visit, the Kurtzke Expanded Disability Status Scale (EDSS) (25) score, and medication information were documented. At the time of sample collection, the EDSS score of acute-stage suffers was assessed by trained physicians. Clinical manifestations, clinical signs, ophthalmic examination, interpreted by experienced ophthalmologists, and magnetic resonance imaging sequences, performed following standard protocols and reported by two neuroradiologists blinded to the research, were also documented to identify lesion location.

### 2.3 Cell-based assay for AQP4-IgG and myelin oligodendrocyte glycoprotein immunoglobulin G detection

Venous blood (5 ml) was mainly collected for serum using blood collection devices that contained separating gel and coagulant accelerator. After that, HEK-293 cells, having been transfected with either human myelin oligodendrocyte glycoprotein or aquaporin-4, were incubated with serum samples isolated from patients and diluted to an appropriate concentration. Subsequently, fluorescently labeled secondary antibodies were added to cells, which had been carefully washed several times with PBS to remove unbound serum components. Finally, fluorescence signals of MOG-IgG and AQP4-IgG were detected and their intensities were determined. This was achieved using flow cytometry and a standard curve of positive control.

### 2.4 Blood routine examination

Venous blood (5 ml) was collected and subsequently deposited in a tube (Sterile K2EDTA, Hebei Xinle Medical Equipment Technology Co.). An automated blood analyzer (UniCel DxH 800 Coulter Cellular Analysis System, Beckman Coulter) was utilized for measurement of blood samples. And then, platelet (PLT)/lymphocyte ratio (PLR) and neutrophil/lymphocyte ratio (NLR) were calculated by dividing the PLT counts (×10^9^/L) by the lymphocyte counts (×10^9^/L) and the neutrophil counts (×10^9^/L) by the lymphocyte counts (×10^9^/L), respectively. The systemic inflammation index (SII) was calculated by multiplying the PLT counts (×10^9^/L) by the neutrophil counts (×10^9^/L) and dividing by the lymphocyte counts (×10^9^/L).

### 2.5 Flow cytometry

Flow cytometry was executed according to the following procedures: First, approximately 2 ml of EDTA-anticoagulated venous blood was drawn from each participant. Then, a volume of 50 µL anticoagulated venous blood was carefully transferred into the base of a tube (BD Trucount™ Tube, BD Biosciences, USA) that was preloaded with fluorescently tagged antibodies specific to CD3, CD4, CD8, CD16, CD19, and CD56, followed by capping, vortexing gently for 5–10 seconds, and incubating for 15 minutes at room temperature in the dark. Afterward, 450 µL of BD FACS™ Lysing Solution (BD Biosciences, USA) was introduced into it, followed by recapping, shaking, and mixing thoroughly. It was subsequently incubated for a further 10 minutes at room temperature with no light. Ultimately, the BD FACSCanto™ II flow cytometer (BD Biosciences, USA) was utilized to identify the cell populations expressing CD3+, CD3+CD4+, CD3+CD8+, CD19+, and CD3-CD16+CD56+ markers. And then, CD4/CD8 ratio (CD4/CD8) was calculated by dividing the CD3+CD4+ T cell counts (/µL) by the CD3+CD8+ T cell counts (/µL).

### 2.6 Statistical analysis

Normality assessment of quantitative variables was conducted using the Kolmogorov-Smirnov test. Data were expressed in the form of means ± standard error of the mean, median (interquartile range, IQR), and proportions, as appropriate. To detect differences between groups whose data passed the normality test, the paired Student’s t-test was applied to paired samples, while the unpaired Student’s t-test was applied to unpaired samples. For the data that failed the normality test, the Mann-Whitney test was conducted to compare unpaired groups, while the Wilcoxon matched test was conducted to compare paired groups. Differences in qualitative variables between groups were calculated using the Chi-square test. Spearman rank correlation was used to calculate correlations among various parameters, with Bonferroni correction applied for multiple tests. The threshold for significance was set at a p value < 0.05. Data analysis was conducted using SPSS 26.0 (IBM, IL, USA) and GraphPad Prism 10.2.0 (GraphPad Inc., CA, USA).

## 3 Results

### 3.1 The fundamental details of included individuals

The study included 76 patients with acute NMOSD, 20 patients with acute MS, 12 patients with acute MOGAD, and 37 age- and sex-matched controls for NMOSD. **Table 1** exhibits an overview of the fundamental details of included individuals.

**Table 1:** Fundamental details of included individuals.

Variables	Acute NMOSD (n = 76)			Acute MS (n = 20)	Acute MOGAD (n = 12)	Control (n = 37)	*p*1	*p*2	*p*3	*p*4	*p*5	*p*6
	Non-DMT-NMOSD (n = 58)	DMT-NMOSD (n = 18)	Total (n = 76)									
Age, median (IQR), years	41 (34–56)	42 (27-56.5)	41 (33.25-56)	31.5 (25.75-35.75)	32.5 (28.5-47.25)	56 (35-60)	0.128	0.080	0.069	0.385	0.005	0.038
Female, n (%)	50 (86.2)	17 (94.4)	67 (88.2)	12 (60)	6 (50)	33 (89.2)	0.670	0.525	0.872	0.345	0.010	0.003
The number of onsets, median (IQR)	2 (1-2.25)	2.5 (2-5.25)	2 (1-3)	2.5 (2-3.75)	1 (1-2)	na	na	na	na	0.001	na	na
The time from last symptom to clinic visit, median (IQR), days	3.5 (2-6.25)	4 (1-5.5)	4 (1.25-6)	4.5 (3-7)	4 (2.25-9.25)	na	na	na	na	0.493	na	na
EDSS score, median (IQR)	5 (3-6)	4.25 (2.375-5.75)	5 (3-6)	2.5 (2-5.625)	3 (2.25-3)	na	na	na	na	0.412	na	na
AQP4-IgG positive or MOG-IgG positive, n (%)	54 (93.1)	16 (88.9)	70 (92.1)	na	12 (100)	na	na	na	na	0.562	na	na
Lesion location												
Optic nerve, n (%)	16 (27.6)	7 (38.9)	23 (30.3)	2 (10)	9 (75)	na	na	na	na	0.362	na	na
Spinal cord, n (%)	40 (69)	10 (55.6)	50 (65.8)	10 (50)	3 (25)	na	na	na	na	0.295	na	na
Area postrema, n (%)	4 (6.9)	2 (11.1)	6 (7.9)	0 (0)	0 (0)	na	na	na	na	0.562	na	na
Brainstem, n (%)	4 (6.9)	2 (11.1)	6 (7.9)	6 (30)	1 (8.3)	na	na	na	na	0.562	na	na
Cerebrum, n (%)	4 (6.9)	1 (5.6)	5 (6.6)	6 (30)	2 (16.7)	na	na	na	na	0.841	na	na
Diencephalon, n (%)	1 (1.7)	0 (0)	1 (1.3)	1 (5)	0 (0)	na	na	na	na	0.575	na	na

NMOSD, neuromyelitis optica spectrum disorder; MS, multiple sclerosis; MOGAD, myelin oligodendrocyte glycoprotein antibody disease; AQP4-IgG, aquaporin-4 immunoglobulin G; MOG-IgG, myelin oligodendrocyte glycoprotein immunoglobulin G; EDSS, Expanded Disability Status Scale; IQR, interquartile range; na, not applicable. *P*1 for Control vs. non-DMT-NMOSD, *p*2 for Control vs. DMT-NMOSD, *p*3 for Control vs. acute NMOSD, *p*4 for non-DMT-NMOSD vs DMT-NMOSD, *p*5 for Control vs. MS, *p*6 for Control vs. MOGAD.

### 3.2 Peripheral blood immune cell profiles in acute NMOSD (including DMT-NMOSD and non-DMT-NMOSD) and control

To tease out peripheral blood immune cell profiles in acute NMOSD, comparative analyses were conducted among non-DMT-NMOSD, DMT-NMOSD, acute NMOSD, and Control. To begin with, there were no marked differences in sex (*p* > 0.05) or age (*p* > 0.05) when comparing acute NMOSD, DMT-NMOSD, and non-DMT-NMOSD to Control individually, as well as when comparing DMT-NMOSD to non-DMT-NMOSD. Moreover, compared with the Control, in acute NMOSD, DMT-NMOSD and non-DMT-NMOSD, white blood cell (WBC), PLT counts, and PLR went up, so did the proportions of CD3+CD8+ T and natural killer (NK) cells; while lymphocyte counts, basophil counts and proportions, CD3+ T cell counts and proportions, CD3+CD4+ T cell counts and proportions, CD19+ B cell counts, and CD4/CD8 showed a downward trend. Notably, these parameters did not differ significantly between DMT-NMOSD and non-DMT-NMOSD.

Furthermore, neutrophil counts, neutrophil proportions, NLR, and SII in acute NMOSD, DMT-NMOSD, and non-DMT-NMOSD were higher than those in Control, while lymphocyte proportions, eosinophil proportions, and eosinophil counts in acute NMOSD, DMT-NMOSD, and non-DMT-NMOSD were lower than those in Control. Attention should be drawn to the fact that in contrast to NMOSD patients without DMT before onset, those in DMT-NMOSD had elevated neutrophil counts, neutrophil proportions, NLR, and SII, while lymphocyte and eosinophil proportions, as well as eosinophil counts, were at lower levels. Overall, although these indicators differed between DMT-NMOSD and non-DMT-NMOSD, they showed the same trend when comparing non-DMT-NMOSD, DMT-NMOSD, and acute NMOSD with Control, respectively.

**Figure 1:**
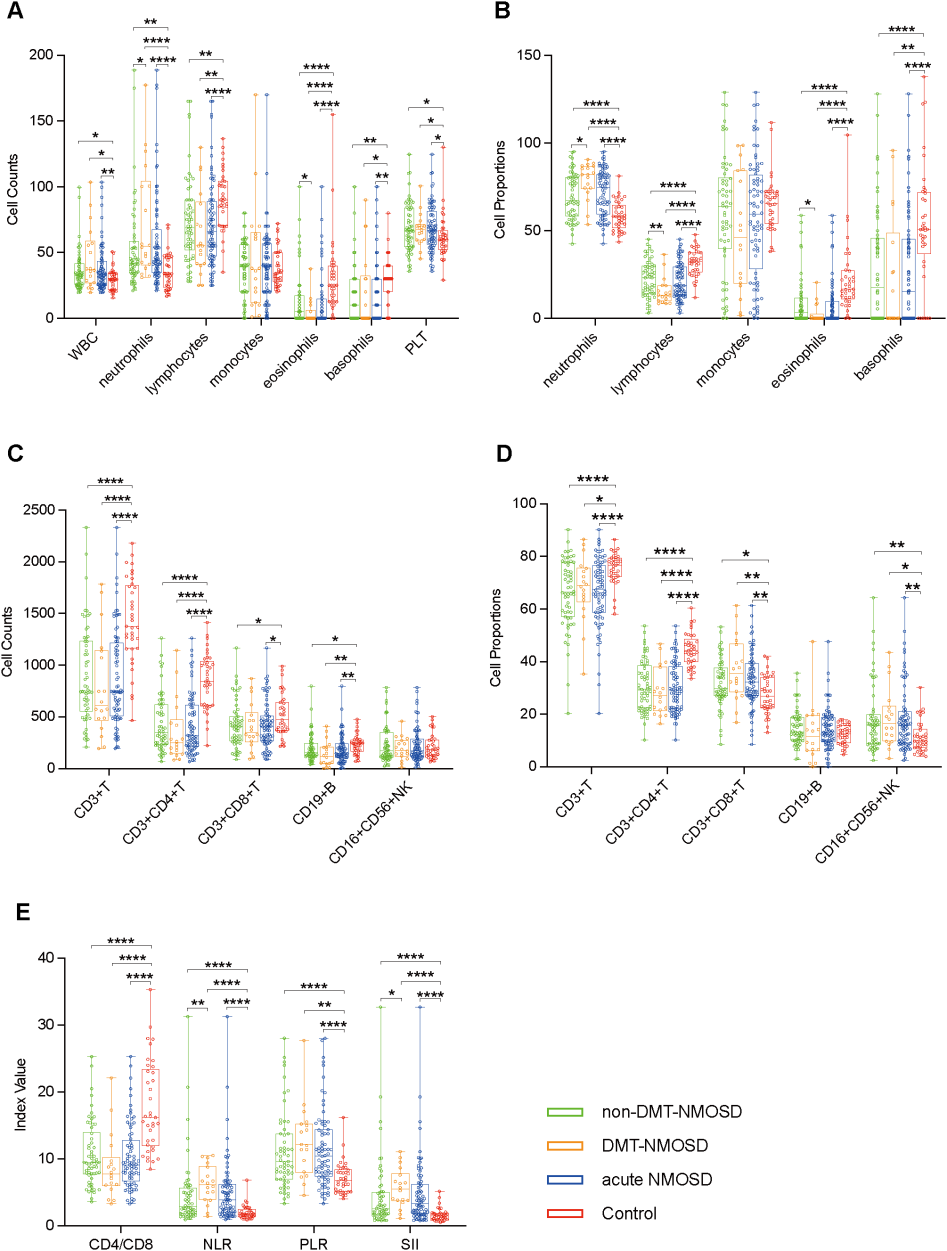
Boxplots depicting comparisons between non-DMT-NMOSD (n = 58) versus Control (n = 37), DMT-NMOSD (n = 18) versus Control, acute NMOSD (n = 76) versus Control, DMT-NMOSD versus non-DMT-NMOSD. **(A)** Comparisons of peripheral blood cell counts. The units of the variables are shown as follows: WBC = ×2×10^8^/L, neutrophils = ×10^8^/L, lymphocytes = ×2×10^7^/L, monocytes = ×10^7^/L, eosinophils = ×4×10^6^/L, basophils = ×10^6^/L, PLT = ×4×10^9^/L. **(B)** Comparisons of peripheral blood cell proportions. The units of the variables are shown as follows: neutrophils = % of WBC, lymphocytes = % of WBC, monocytes = ‰ of WBC, eosinophils = ‰ of WBC, basophils = ‰ of WBC. **(C)** Comparisons of peripheral blood lymphocyte counts. The units of all parameters are/µL. **(D)** Comparisons of peripheral blood lymphocyte proportions. The units of all parameters are % of lymphocytes. **(E)** Comparisons of ratio-based indices. Boxplots correspond to the median with interquartile range, the lower and upper whiskers extend from the hinge to the lowest and highest values (respectively), and the dots represent individual values. *P<0.05, **P<0.01, ***P<0.001, ****P<0.0001.

In addition, CD3+CD8+ T cell counts were decreased when comparing acute NMOSD and non-DMT-NMOSD to Control, otherwise, no notable differences were observed for CD3+CD8+ T cell counts when comparing non-DMT-NMOSD and Control to DMT-NMOSD. Also, no significant differences were noted in monocyte counts, monocyte proportions, CD16+CD56+ NK cell counts, and CD19+ B cell proportions when comparing DMT-NMOSD versus non-DMT-NMOSD, acute NMOSD versus Control, DMT-NMOSD versus Control, and non-DMT-NMOSD versus Control (**Figures 1A–E**).

### 3.3 Peripheral blood immune cell profiles in paired acute NMOSD and remission NMOSD

**Figure 2:**
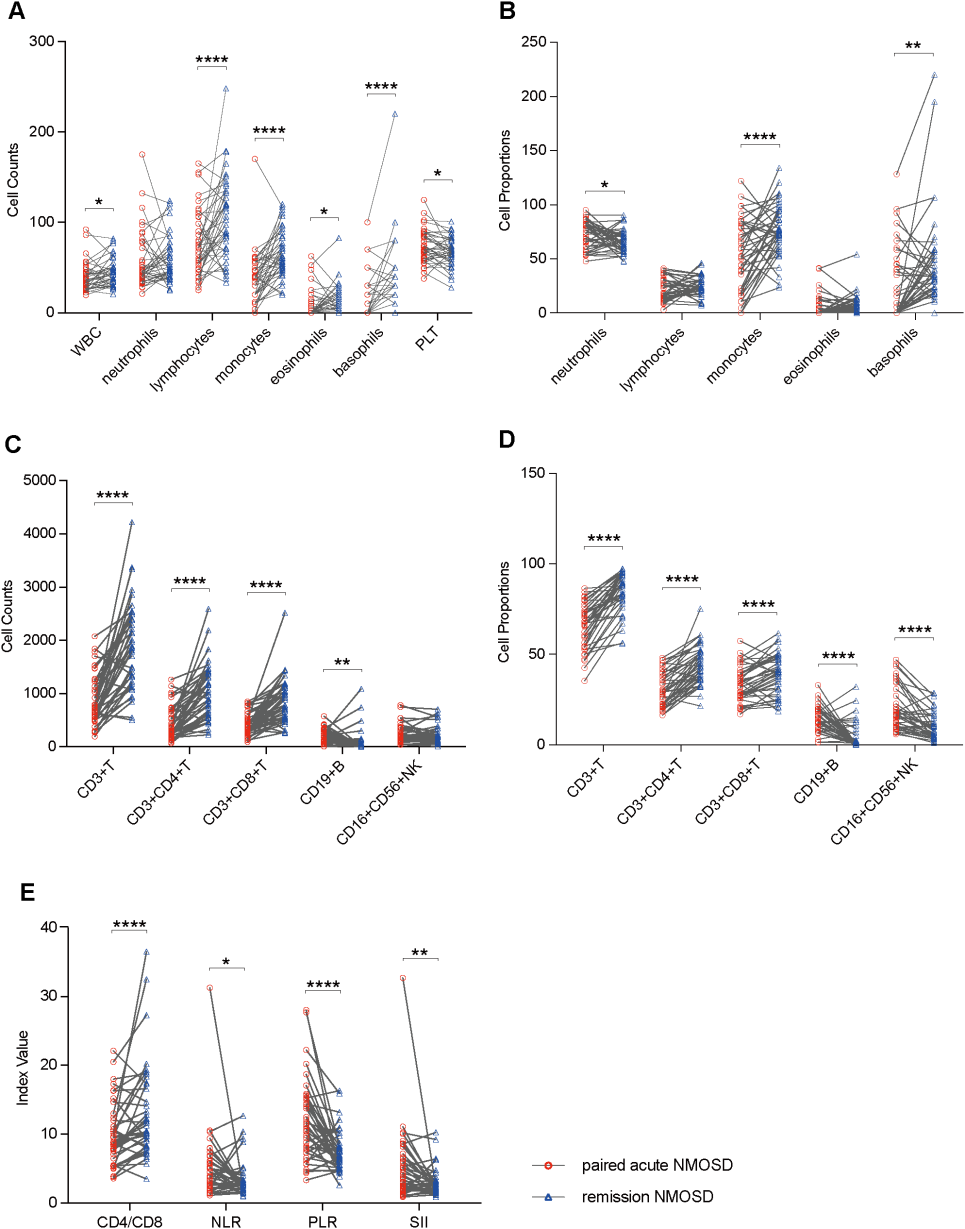
Boxplots illustrating comparisons between remission NMOSD (n = 42) and paired acute NMOSD (n = 42): **(A)** Comparisons of peripheral blood cell counts. **(B)** Comparisons of peripheral blood cell proportions. **(C)** Comparisons of peripheral blood lymphocyte counts. **(D)** Comparisons of peripheral blood lymphocyte proportions. **(E)** Comparisons of ratio-based indices. *P<0.05, **P<0.01, ****P<0.0001.

To assess the dynamics of peripheral blood immune cell profiles in different phases of the same case, paired blood samples from 42 patients in the acute phase and in remission were obtained and measured. Compared to remission, acute-phase NMOSD showed elevated PLT counts, neutrophil proportions, CD19+ B cell counts and proportions, CD16+CD56+ NK cell proportions, NLR, PLR, and SII, but reduced WBC counts, lymphocyte counts, monocyte counts and proportions, eosinophil counts, basophil counts and proportions, CD3+ T cell counts and proportions, CD3+CD4+ T cell counts and proportions, CD3+CD8+ T cell counts and proportions, and CD4/CD8. Additionally, neutrophil counts, lymphocyte proportions, eosinophil proportions, and CD16+CD56+ NK cell counts were similar in the acute phase and remission (**Figures 2A–E**).

### 3.4 Peripheral blood immune cell profiles in rituximab group, inebilizumab group, and CIT group

**Figure 3:**
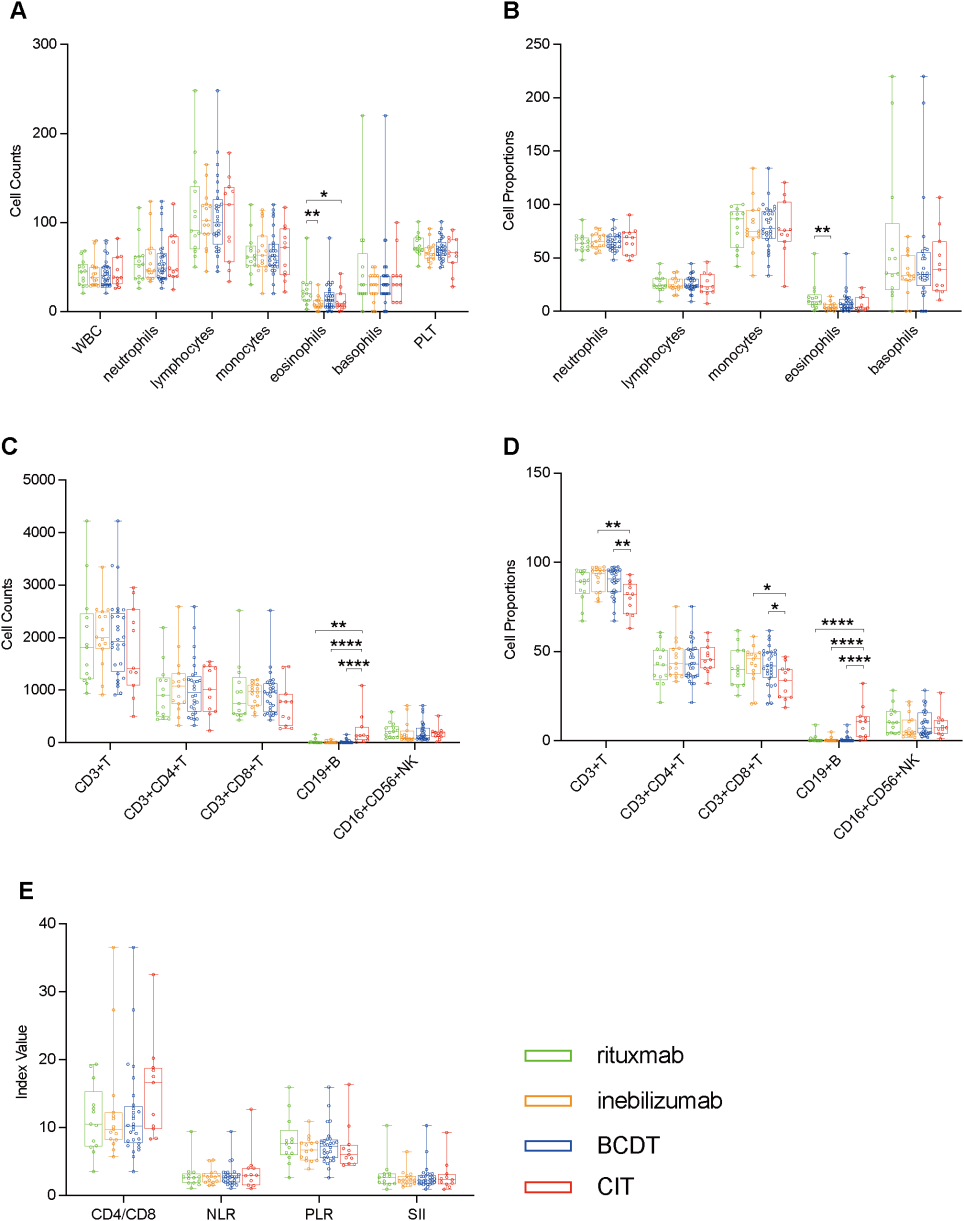
Boxplots illustrating pairwise comparisons between BCDT group (n = 28) and CIT group (n = 11), between inebilizumab group (n = 15) and CIT group, between rituximab group (n = 13) and CIT group, and also directly between inebilizumab group and rituximab group using the unpaired Student’s t-test or the Mann-Whitney test: **(A)** Comparisons of peripheral blood cell counts. **(B)** Comparisons of peripheral blood cell proportions. **(C)** Comparisons of peripheral blood lymphocyte counts. **(D)** Comparisons of peripheral blood lymphocyte proportions. **(E)** Comparisons of ratio-based indices. *P<0.05, **P<0.01, ****P<0.0001.

To elucidate the differential impacts of distinct pharmacological agents on peripheral blood immune cell profiles, pairwise comparisons were made between BCDT group and CIT group, between inebilizumab group and CIT group, between rituximab group and CIT group, and also directly between inebilizumab group and rituximab group using the unpaired Student’s t-test or the Mann-Whitney test. The median age of the BCDT group was younger than that of the CIT group (median age: 58 vs. 38, *p* = 0.038). However, no significant differences in age and sex were observed between the inebilizumab group and the CIT group, the rituximab group and the CIT group, as well as between the inebilizumab group and the rituximab group (*p* > 0.05). Additionally, there was no significant difference in gender distribution between the BCDT group and the CIT group (*p* > 0.05). Compared with the CIT group, the BCDT group exhibited a decrease in CD19+ B cell level and an increase in the percentages of CD3+ T cells and CD3+CD8+ T cells. Compared with the use of CIT alone, after rituximab treatment, not only did CD19+ B cell level decrease, but eosinophil counts also increased. Moreover, compared with the use of CIT alone, after inebilizumab treatment, not only did CD19+ B cell level decrease, but also the ratios of CD3+ T cells and CD4+CD8+ T cells increased. Furthermore, the number and proportions of eosinophils in rituximab group exhibited an upward trend in comparison to inebilizumab group. No notable bias was observed between the groups for other parameters of peripheral blood immune cells (**Figures 3A–E**).

### 3.5 Peripheral blood immune cell profiles in non-DMT-NMOSD, acute MS, and acute MOGAD

**Figure 4:**
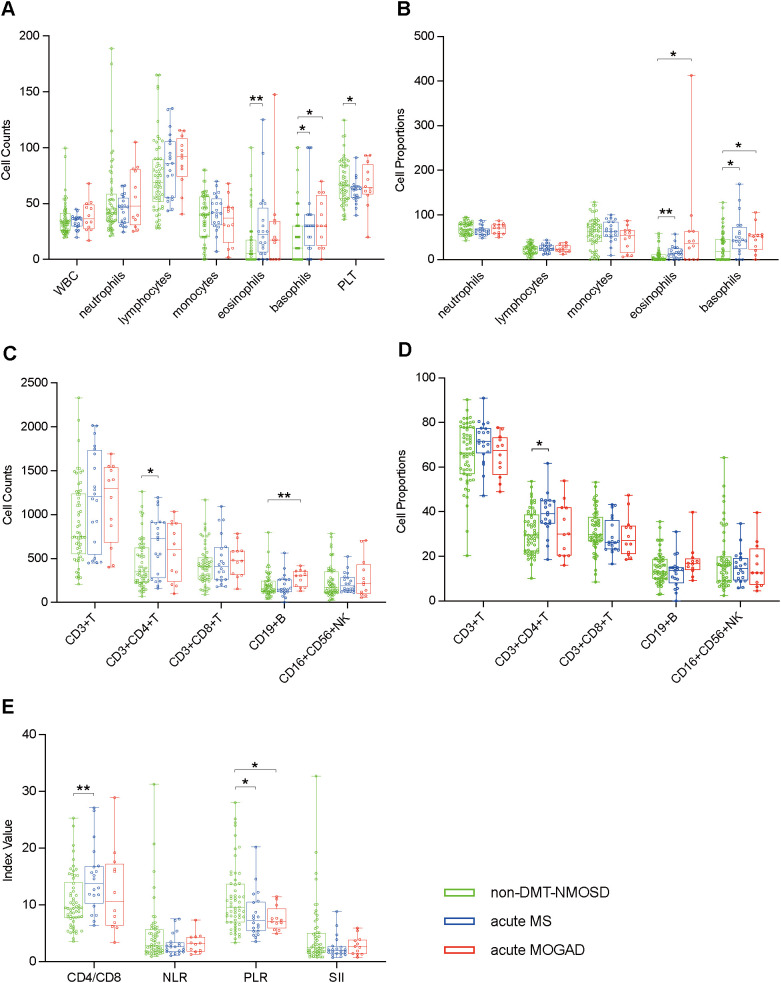
Boxplots illustrating comparisons between non-DMT-NMOSD (n = 58) versus acute MS (n = 20) and non-DMT-NMOSD versus acute MOGAD (n = 12) using the unpaired Student’s t-test or the Mann-Whitney test: **(A)** Comparisons of peripheral blood cell counts. **(B)** Comparisons of peripheral blood cell proportions. **(C)** Comparisons of peripheral blood lymphocyte counts. **(D)** Comparisons of peripheral blood lymphocyte proportions. **(E)** Comparisons of ratio-based indices. *P<0.05, **P<0.01.

To understand the differences in peripheral blood immune cell profiles of NMOSD with MS and NMOSD with MOGAD, we compared the peripheral blood immune cell profiles of non-DMT-NMOSD with acute MS and acute MOGAD, respectively, using the unpaired Student’s t-test or the Mann-Whitney test. Individuals in non-DMT-NMOSD were older, with a median age of 41 compared to 31.5 (acute MS, *p* = 0.002) and 32.5 (acute MOGAD, *p* = 0.04). The female population was larger in non-DMT-NMOSD (88.2%) than in acute MS (60%, *p* = 0.012) and acute MOGAD (50%, *p* = 0.004). Moreover, patients in non-DMT-NMOSD group had significantly higher levels of PLT counts and PLR as well as lower levels of CD4/CD8, eosinophils, basophils, and CD3+CD4+ T cells in both counts and proportions than patients with acute MS. Furthermore, eosinophil proportions, basophil counts, basophil proportions, and CD19+ B cell counts were decreased when comparing non-DMT-NMOSD with acute MOGAD, however, PLR was elevated. In addition, no differences regarding other indicators of peripheral blood immune cells were identified between the groups (**Figures 4A–E**).

### 3.6 Correlation matrices of various parameters in the paired acute NMOSD, the remission NMOSD, and control

**Figure 5:**
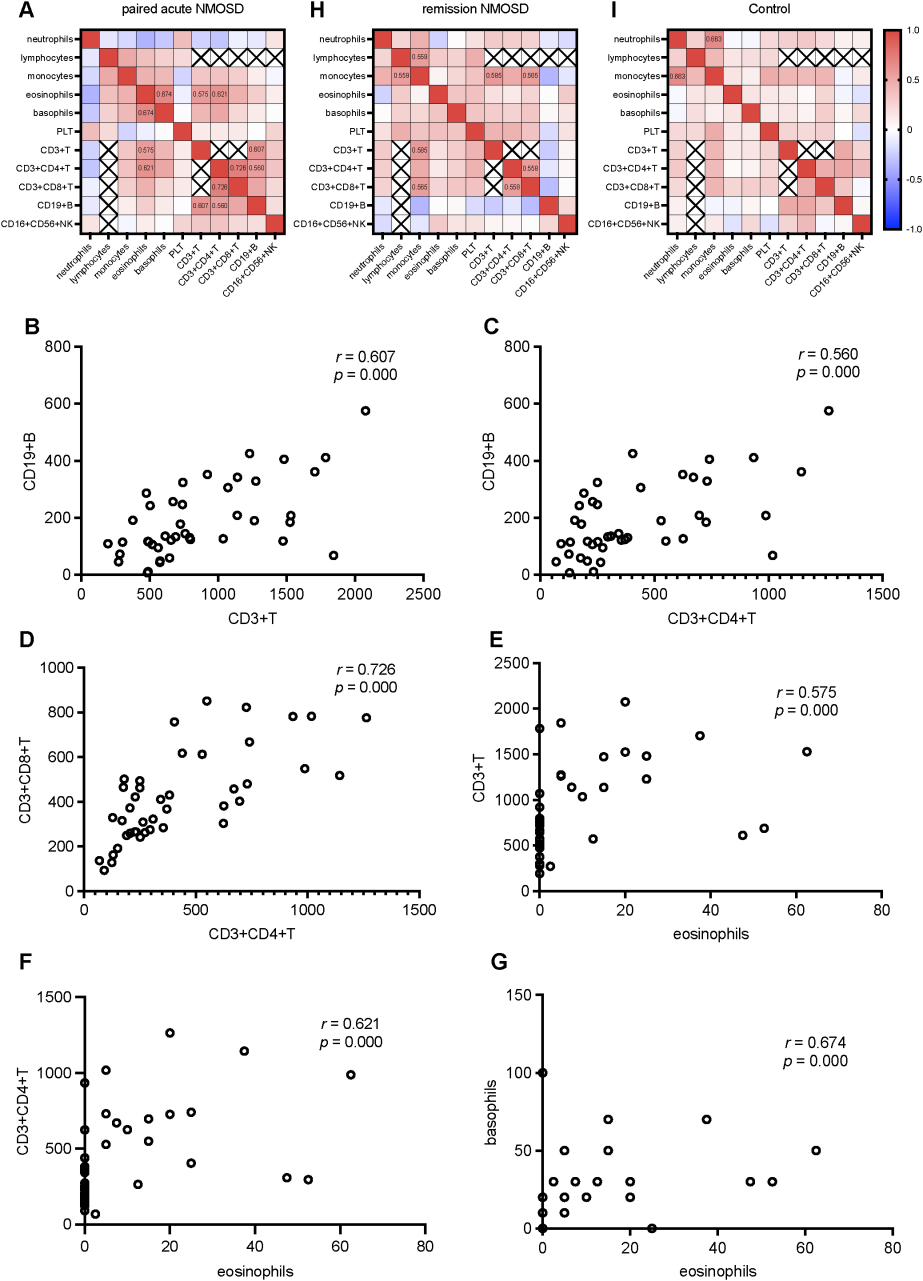
Correlation analysis of various parameters in the paired acute NMOSD, the remission NMOSD, and Control: **(A)** Correlation heatmap of various parameters in the paired acute NMOSD. **(B)** CD19+ B cell counts positively correlated with CD3+ T cell counts. **(C)** CD19+ B cell counts positively correlated with CD3+CD4+ T cell counts. **(D)** CD3+CD4+ T cell counts positively correlated with CD3+CD8+ T cell counts. **(E)** CD3+ T cell counts positively correlated with. eosinophil counts. **(F)** CD3+CD4+ T cell counts positively correlated with. eosinophil counts. **(G)** Basophils counts positively correlated with. eosinophil counts. **(H)** Correlation heatmap of various parameters in the remission NMOSD. **(I)** Correlation heatmap of various parameters in Control.

In order to explore the interaction network among different immune parameters, we conducted a correlation analysis of the indices of the absolute numbers of various immune cells. Since the WBC counts are the sum of various peripheral immune cell counts, their correlations with individual peripheral immune cell counts were not analyzed. Similarly, as B cells, T cells, and NK cells are subtypes of lymphocytes, and CD3+CD4+ T cells and CD3+CD8+ T cells are subtypes of CD3+ T cells, we did not investigate the correlations between CD19+ B cell counts, CD16+CD56+ NK cell counts, CD3+ T cell counts and lymphocyte counts, nor those between CD3+CD4+ T cell counts, CD3+CD8+ T cell counts and CD3+ T cell counts. During the acute phase of NMOSD, positive correlations were observed between CD3+CD4+ T cell counts and CD3+CD8+ T cell counts, as well as between CD3+CD4+ T cell counts and CD19+ B cell counts. Additionally, CD3+ T cell counts was positively correlated with CD19+ B cell counts. Moreover, eosinophil counts showed positive correlations with CD3+ T cell counts, CD3+CD4+ T cell counts, and basophil count s, respectively (**Figures 5A–G**). In the remission phase of NMOSD, positive correlations were found between monocyte counts and lymphocyte counts, CD3+ T cell counts, CD3+CD8+ T cell counts, as well as between CD3+CD4+ T cell counts and CD3+CD8+ T cell counts (**Figure 5H**). In the Control, only a positive correlation between monocyte counts and neutrophil counts was observed (**Figure 5I**).

## 4 Discussion

As an autoimmune disease, NMOSD is probably triggered by a dysregulated immune system. Historically, researches on NMOSD have predominantly focused on B cells, T cells, and a certain subpopulation of them, roles of innate immune cells in NMOSD have largely been ignored (5–9). The present real-world study, based on Han Chinese from northern China, provided a systematic and comprehensive description of peripheral blood immune cell profiles of NMOSD across different disease states, after BCDT, and also compared with MS and MOGAD, two diseases that are easily confused with NMOSD. The results demonstrated widespread changes in peripheral blood immune cell abundance in acute NMOSD, suggesting that the pathogenesis involves not only B cells and T cells but also other peripheral blood immune cells.

Except for a lower number of onsets in the non-DMT-NMOSD compared to the DMT-NMOSD, no differences were observed in demographic or other clinical features between the two groups. This discrepancy in the number of onsets might be explained by the inclusion of 22 first-onset patients in the non-DMT-NMOSD.

Except for CD3+CD8+ T cells, the majority of peripheral blood immune cells showed the same variation trend, when DMT-NMOSD versus Control, non-DMT-NMOSD versus Control, and acute NMOSD versus Control. This suggests that the onset of NMOSD depends primarily on whether the balance of the immune system is disrupted rather than whether DMT is received. Notably, based on the above situation, in the discussion section, we do not explain the changes of these parameters between non-DMT-NMOSD and Control as well as DMT-NMOSD and Control respectively, but only summarize between acute NMOSD and Control.

We observed that WBC counts in acute NMOSD were inferior to those in remission NMOSD, but elevated in acute NMOSD patients compared to controls. The fact that WBC contain a variety of subtypes with distinct functions may explain the conflicting trend in WBC counts.

Our investigation revealed that both neutrophil counts and proportions in acute NMOSD patients were significantly higher compared to controls. Additionally, during the acute phase, neutrophil proportions in these patients were notably increased compared to their levels during remission. Recently, a Japanese study also demonstrated a significant increase in neutrophil counts when comparing NMOSD with Control (26). It is worth noting that our study is not a duplicate of this study. Our study compared acute NMOSD patients with controls and further compared acute NMOSD patients with paired remission NMOSD patients. Furthermore, our study analyzed not only neutrophil counts but also neutrophil proportions. According to previous studies, neutrophils facilitated NMOSD pathogenesis by promoting B cell and T cell responses, damaging the blood-brain barrier and astrocyte, and activating the complement system (27–30).

It has been reported that NMOSD monocytes were susceptible to activation, produced more inflammatory cytokines, and expressed more inflammatory surface molecules, implying that monocytes might play a pathogenic role in NMOSD (31). However, our results did not show an increased abundance of monocytes in acute NMOSD. This suggests that the function of monocytes in the pathogenesis of NMOSD lies in augmenting inflammatory function rather than in elevating cell abundance.

Eosinophil counts and proportions were lower in acute NMOSD patients than in controls and remission-phase NMOSD patients. Eosinophils were detected in active lesions of NMOSD patients during the initial phase, as well as in the cerebrospinal fluid during periods of relapse and remission (32). The mechanisms driving elevated CNS eosinophils alongside reduced blood levels remain unclear, and we speculate whether it is due to the migration of eosinophils into the CNS.

Our findings indicated reduced basophil counts and proportions in acute NMOSD in comparison to Control and remission NMOSD. The function of basophils within the context of NMOSD has yet to be fully understood. Nevertheless, investigations into other autoimmune disorders have shed light on their significance. For instance, in patients suffering from systemic lupus erythematosus, basophils were observed to promote the production of B cell autoantibodies and the differentiation of Th17 cells. Interestingly, a deficiency of basophils was noted in such patients, potentially as a result of the migration of these cells to the lymph nodes (33).

Lymphocyte counts were lower in acute NMOSD than in both controls and remission, while their proportions were reduced only versus controls. Lymphocyte subsets are further characterized as follows.

Acute NMOSD patients and controls had similar CD19+ B cell proportions, which corresponded approximately to a recent research performed by Jiang et al. (34). Their research findings signified that the proportion of B lymphocytes in NMOSD sufferers was steady, in contrast to a distinct augmentation in the proportions of plasma cells and memory B lymphocytes (34). In our research, the diminished CD19+ B cell counts in acute NMOSD in contrast to the Control may stem from the decreased lymphocyte counts. However, both the quantity and percentages of CD19+ B cells increased in acute NMOSD as opposed to the remission phase of NMOSD. This was in line with the fact that BCDT notably cut down the recurrence of NMOSD (11, 12).

CD3+ T cell counts and proportions of acute NMOSD patients were reduced, both compared to controls and remission NMOSD patients. Furthermore, we found that CD3+CD4+ T cell proportions dropped in acute NMOSD compared to Control, which was in agreement with previous findings (34, 35), and we found that CD3+CD4+ T cell counts also dropped. Moreover, CD3+CD4+ T cell levels in acute NMOSD dropped compared to remission NMOSD. Traditionally, CD3+CD4+ T cells have been recognized as a contributor to the pathogenesis of NMOSD (6, 9). The root of the diminished T cell count and ratios in acute NMOSD calls for additional probing. CD3+CD8+ T cells contribute to NMOSD pathogenesis (7, 8). In our study, CD3+CD8+ T cell proportions of acute NMOSD patients elevated compared to controls, which was similar to a recent study using single-cell sequencing and flow cytometry (34), while CD3+CD8+ T cell counts decreased. The contradictory trends in CD3+CD8+ T cell counts and proportions may be associated with the reduction of lymphocyte counts. The proportions of CD16+CD56+ NK cells showed a distinct elevation in acute NMOSD in contrast to Control and remission NMOSD. Through manifesting CD16 and complement receptors, triggering antibody-dependent cellular cytotoxicity, and continuous autoantibody production, NK cells could trigger the onset of NMOSD (36).

The pro-inflammatory functions of PLT, which have been gradually recognized, included leading the cells within the innate immune framework to alter the inflammatory response, supporting the surveillance of adaptive immune system, and influencing both the production of antibodies and the polarisation state of T cells (37). Hence, we tried to evaluate PLT alterations in NMOSD. As per our expectations, an elevated level of PLT counts was observed in acute NMOSD compared to both Control and remission NMOSD.

Our research delved into how two B cell depletion medications, namely rituximab and inebilizumab, affected peripheral blood immune cell profiles of NMOSD patients, which, as far as we are aware, has not been explored. In comparison to those who received CIT alone, patients administered with rituximab or inebilizumab manifested a marked reduction in both the quantity and proportions of CD19+ B cells. In addition, there was an elevation in eosinophil counts subsequent to rituximab treatment. The CD3+T cell and CD3+CD8+ T cell ratios in both the BCDT group and the inebilizumab group showed an upward trend compared to the CIT group. Since the immune system constitutes a dynamic and unified web of interactions, BCDT induces changes in immune cells besides B cells through complex immune regulation. A trial demonstrated that despite the extensive depletion of B cells, no prominent elevation in the susceptibility to tumors and infections was observed subsequent to the administration of inebilizumab (38). Perhaps the relative increase in CD3+ T cell and CD3+CD8+ T cell proportions by inebilizumab can explain this phenomenon. Rituximab and inebilizumab are an anti-CD20 and an anti-CD19 monoclonal antibody, respectively. Given that CD19 has a broader expression pattern on B cells compared to CD20, inebilizumab theoretically depletes B cells more extensively. However, we did not observe significantly lower CD19+ B cell levels in inebilizumab group compared to rituximab group. B cell levels in both inebilizumab and rituximab groups were profoundly depressed, which may explain the lack of significant differences in B cell counts between them. Additionally, our findings indicated that eosinophil counts were greater in rituximab cohort as opposed to the inebilizumab one. Given the part eosinophils play in fueling the disease progression within NMOSD, it is necessary to conduct further extended follow-up investigations to more comprehensively fathom and contrast the effectiveness of these two medications.

Considering the abnormal counts of various immune cells during the onset of NMOSD, we further investigated the composite inflammatory indices, including CD4/CD8, NLR, PLR, and SII. These ratio-based indices have been widely recognized in recent years as integrative biomarkers of systemic immune inflammatory responses (39, 40). Compared with single indicators, they can more comprehensively reflect the immune status of the body. The levels of CD4/CD8 decreased both when comparing acute NMOSD patients with the control group and when comparing paired acute NMOSD and remission NMOSD patients. As previously mentioned, both CD3+CD4+ T cell counts and CD3+CD8+ T cell counts decreased in NMOSD patients. This index can reflect the dynamic changes of CD3+CD4+ T cells and CD3+CD8+ T cells in NMOSD patients, suggesting that during the acute phase of NMOSD, the decrease in CD3+CD4+ T cells is more significant than that of CD3+CD8+ T cells. In addition, the correlation analysis showed that there was a positive correlation between the numbers of CD3+CD4+ T cells and CD3+CD8+ T cells in the acute phase of NMOSD (*r* = 0.726). Although there was also a positive correlation in the remission phase (*r* = 0.558), the correlation was less significant than that in the acute phase, and this correlation was not present in the control group. This suggests that the interaction between CD3+CD4+ T cells and CD3+CD8+ T cells may play an important role in the pathogenesis of NMOSD. As composite immune indices comprehensively reflecting the innate immune process, NLR, PLR, and SII increased in the acute phase of NMOSD, whether compared with the control group or the remission NMOSD group, which indicates that the innate immune process is involved in the pathogenesis of NMOSD. When comparing the rituximab group, the inebilizumab group, and the BCDT group with the CIT group respectively, and when comparing the rituximab group with the inebilizumab group, there were no differences in the three composite inflammatory indices, suggesting that the patients in the remission phase treated with different drugs are in a similar state of immune balance.

In the acute phase of NMOSD, a positive correlation was observed between CD3+CD4+ T cell counts and CD19+ B cell counts. Notably, this correlation was absent in the remission phase of NMOSD and in the control group, strongly suggesting a unique interaction between CD4+ T cells and CD19+ B cells that is specifically involved in the pathogenesis of NMOSD. This observation highlights the potential role of the interplay between these two immune cell populations in triggering the acute onset of the disease, which is distinct from the disease remission state and the normal immune status represented by the control group. Additionally, our study revealed positive correlations between eosinophil counts and multiple immune cell counts, including CD3+ T cell counts, CD3+CD4+ T cell counts, and basophil counts. These correlations imply that eosinophils may contribute to NMOSD relapses by interacting with various immune cells, thereby exerting their functional roles. It is likely that in future research, we could direct our attention to the specific mechanisms through which eosinophils participate in the onset and development of NMOSD. In the remission phase of NMOSD, positive correlations were identified between monocyte counts and multiple immune cell counts, such as lymphocyte counts, CD3+ T cell counts, and CD3+CD8+ T cell counts. These associations suggest that monocytes participate in the regulation of disease remission by interacting with diverse immune cells. Notably, the current study only demonstrates numerical correlations among these immune cell populations. Further investigations are warranted to elucidate the specific molecular and cellular mechanisms underlying these interactions, which will be crucial for a comprehensive understanding of the immunopathogenesis of NMOSD and the development of targeted therapeutic strategies.

It was shown in our study that the amount and ratio of CD3+CD4+ T lymphocytes in MS sufferers exhibited a relatively higher state compared to that in NMOSD sufferers, which corresponds to the fundamental function that the cells fulfill within the context of MS (41). We also found the levels of eosinophils and basophils as well as CD4/CD8 decreased, as well as PLT counts and PLR increased in NMOSD compared to MS. Moreover, compared with MOGAD patients, NMOSD patients had fewer basophil counts, eosinophil ratio, basophil ratio, and CD19+ B cell counts, and elevated PLR. Although the mechanisms underlying these differences remain unclear, these distinctions suggest that NMOSD, MS, and MOGAD are distinct diseases. Further elucidation of the potential mechanisms will enhance our understanding and facilitate a clearer differentiation between these CNS demyelinating disorders.

There were some limitations in our study. First of all, the study population was all Chinese Han from northern China, so the results of the study might be influenced by race and region. Second, as NMOSD and MOGAD belong to rare disease categories, our study had a relatively small sample count. Third, consistent with epidemiological data (42), NMOSD, MS, and MOGAD patients included in this study differed in terms of sex ratio and age distribution. Accordingly, the findings regarding the differences between them might be influenced by sex and age. Finally, the underlying mechanisms of peripheral blood immune cell changes remained unclear.

Summarizing, our study elaborates on the widespread alterations in peripheral blood immune cell profiles of NMOSD, highlighting that not only B cells and T cells but also other immune cells are pivotal to the onset and recurrence of NMOSD. We emphasize that BCDT affects not only B cells but also other peripheral blood immune cells. Also, we point out that following treatments with diverse B cell depleting drugs, peripheral blood immune cell profiles exhibit differential alterations. Additionally, we note that peripheral blood immune cell profiles in NMOSD are different from those in MS and MOGAD. Our findings provide clues to comprehensively understand the abnormality of complex immune networks in NMOSD, distinguish NMOSD from other CNS demyelinating diseases, and search for more effective and safe therapeutic targets.

## Data Availability

The original contributions presented in the study are included in the article/supplementary material. Further inquiries can be directed to the corresponding author.
